# Dynamics of moisture diffusion and adsorption in plant cuticles including the role of cellulose

**DOI:** 10.1038/s41467-021-25225-y

**Published:** 2021-08-19

**Authors:** E. C. Tredenick, G. D. Farquhar

**Affiliations:** ARC Centre of Excellence in Translational Photosynthesis, Division of Plant Science, Research School of Biology, The Australian National University, Canberra, ACT Australia

**Keywords:** Permeation and transport, Plant physiology, Applied mathematics

## Abstract

Food production must increase significantly to sustain a growing global population. Reducing plant water loss may help achieve this goal and is especially relevant in a time of climate change. The plant cuticle defends leaves against drought, and so understanding water movement through the cuticle could help future proof our crops and better understand native ecology. Here, via mathematical modelling, we identify mechanistic properties of water movement in cuticles. We model water sorption in astomatous isolated cuticles, utilising three separate pathways of cellulose, aqueous pores and lipophilic. The model compares well to data both over time and humidity gradients. Sensitivity analysis shows that the grouping of parameters influencing plant species variations has the largest effect on sorption, those influencing cellulose are very influential, and aqueous pores less so but still relevant. Cellulose plays a significant role in diffusion and adsorption in the cuticle and the cuticle surfaces.

## Abstract (web summary)

The plant cuticle provides a barrier between internal leaf tissues and the environment. Here the authors develop a mathematical model of water movement through the cuticle and describe a prominent role for cellulose in controlling the dynamics of moisture diffusion and adsorption.

## Introduction

Food production may need to increase by 70% in the next 30 years, in order to feed the world’s estimated 9.7 billion people^1^, and water loss from plants may hold one key, particularly in a time of changing climate. The plant cuticle is an important layer that covers much of the aerial parts of the plant, including the leaves, fruits and non-woody stems. The cuticle is a largely water-proof outer layer that forms the final defence of leaves in drought and at night, where stomata are closed and water loss needs to be kept to a minimum. In the plant cuticle, the mechanisms of transport including adsorption and diffusion of water are, to date, poorly understood. Understanding the transport mechanisms of water can inform a wide range of scenarios from foliar agrochemical application, plant biology and horticulture.

Several aspects of moisture transport in cuticles have been discovered over the past three decades, including salts on the cuticle surface that may lead to microscopic leaf wetness^2,3^ (Tredenick, E. C., Stuart-Williams, H. & Enge, G., 2021, Materials on plant leaf surfaces are deliquescent in a variety of environments, unpublished), the water sorption capacity of cellulose^4^ and aqueous pores^5,6^. Moisture sorption in plant cuticles is important to consider and can influence the formation of cracks, leading to issues with food production of fruits and mechanical properties of the plant cuticle^7,8^. An experimental and theoretical approach has shown that the small fluxes associated with the cuticle are important to include in leaf gas exchange experiments. The work studied several plant species and a drought condition and found that, under certain conditions, cuticular transpiration was an important parameter to include in the calculations^9^.

Relative humidity (RH) has a large influence over the weight gain due to water sorption, and penetration of hydrophilic ionic agrochemicals, such as calcium chloride (CaCl_2_), across the cuticle^3,6,10^, and so the degree of cuticle hydration is important. Humectants and non-ionic surfactants have been shown to influence the hydration of the cuticle^10,11^. Increasing RH can significantly increase penetration of ionic agrochemicals through plant cuticles, and the mechanisms causing this increase are hydration of the cuticle, water adsorption to the aqueous pore walls^6,12^ and water adsorption due to hygroscopic salts that may exist both in situ on the leaf surface (Tredenick, E. C., Stuart-Williams, H. & Enge, G., 2021, Materials on plant leaf surfaces are deliquescent in a variety of environments, unpublished) and included in the spray formulation^3^. Many mechanisms play an important role in water transport, though RH and the mechanisms involved in plant species variations are among the most influential^3^. Within the cuticle, both bound and free water are present^13^ and we refer to the general process of water travelling into the cuticle as *penetration* or *transport* and the general process where water changes the weight of the cuticle as *sorption*, as there are several specific processes involved in these general processes, such as diffusion (passive Fickian), adsorption (binding) and desorption (unbinding).

Foliar water uptake may play an important ecophysiological role for the plant during drought conditions, though the mechanisms of penetration are not yet fully defined. Foliar water uptake for the plant may be both a cost and a benefit, and a balance is required over the plant’s lifetime. To date, across the phylogeny, the majority of plant species have been found to have the capacity for foliar water uptake^14^. Water may be present on the leaf surface due to rain, dew, high humidity or hygroscopic salts^3^. Foliar water use of a coastal redwood has been characterised and fog suppresses water loss from leaves, ameliorating daily water stress^15^. Leaf wetting may not only play an important role for plants now, but may be increasingly important in the future due to our changing climate, challenging the plant in novel ways^14^.

The importance of a mechanistic mathematical model has been noted previously in the literature^3,16^, and creating models to describe moisture transport processes will improve our understanding of the governing mechanisms. Three mechanistic plant cuticle models have been previously developed^3,17,18^, focusing on an aqueous solution of hydrophilic ionic (CaCl_2_) agrochemical and lipophilic surfactant penetration into the isolated astomatous plant cuticle, applied as a droplet. The models include mechanisms of ion binding and evaporation with hygroscopic water absorption, along with the ability to vary the active ingredient concentration and type, surfactant formulation, RH and plant species. Water penetration was included in the model but validation for water diffusion was not conducted, choosing to validate the CaCl_2_ penetration data as this was the focus. These models form the basis of the current work. We aim to characterise the surface properties and transport pathways of water in isolated astomatous cuticles using mechanistic mathematical modelling techniques and to validate the model with well-known experimental data. The model will account for RH, temperature, sorption of water by cellulose and the ability to model a variety of plant species. The model will be more predictive and less reliant on the need to perform experiments a priori.

The plant cuticle is a structure that is considered the rate-limiting barrier to agrochemical diffusion through plant leaves. It forms a protective layer that is modified by the environment and regulates water loss^19,20^. The plant cuticle is a porous, highly heterogeneous structure that varies between species and individuals in thickness, chemical composition, adaxial and abaxial cuticles, outside and inside surfaces and abundance and arrangement of structures, such as aqueous pores, trichomes, stomata and waxes^21^.

Aqueous pores are dynamic structures within the cuticle that form only in the presence of water^6^ and have been visualised across the cuticle surface but are more concentrated in and around the bases of trichomes and stomata^5,22^. The maximum pore radius varies significantly between plant species, with estimates of 0.3–2.12 nm^17,23^. Aqueous pores must not be confused with cracks or permanent and macroscopic pores^6,24^. Hydrophilic ionic agrochemicals penetrate the plant cuticle exclusively through aqueous pores via diffusion^25^. Water sorption to polar domains leads to swelling of the cutin polymer. The chemical nature of polar pathways can be reasonably speculated on: non-esterified carboxyl and/or hydroxyl groups of cutin monomers or wax molecules could contribute. Alternatively, if polar carbohydrates extended from epidermal cell wall through the cuticle to the exterior, they could form the polar pathway^4,26^. Lipophilic compounds travel through the cuticle in the lipophilic pathway exclusively, by jumping into voids or defects that arise due to molecular motion by the polymer segments or chains, characterised as a three-step process of entering the cuticle via the cuticular lipids, diffusion across the cuticular membrane. The lipophilic pathway is more dependent on temperature and the presence of waxes, when compared to penetration in aqueous pores^6,27,28^.

Water can utilise both the aqueous and lipophilic pathways within the cuticle, as it is a small, uncharged, but polar molecule^5^. Water molecules within aqueous pores can either diffuse as free molecules^6^ or attach to pore walls by adsorption^12^. The share of the aqueous pathway or lipophilic pathway to water penetration in the plant cuticle is currently unknown. However, we can reasonably speculate that this contribution will vary depending on the plant species, environmental conditions and growth conditions. The contribution of each pathway may also be governed by the method of cuticle isolation and chemical treatments, and in the context of agrochemical penetration, pre-treatments and applied droplet chemical composition, including surfactants^4,11,18,29^. One study^5^ found that water transport was 2.8 times higher in the aqueous pathway than the lipophilic pathway of astomatous isolated cuticles, with significant variation between species.

The plant cuticle contains polar polysaccharides, such as crystalline cellulose, hemicellulose and lignins. Polysaccharides contribute 14–28% of the dry weight of isolated cuticles^4,8^. For example, Chamel et al.^4^ found that the contribution to the total water sorption in a cuticle is attributed 67% to polysaccharides, 32% to cutin and 1% to waxes. Although the weight of polysaccharides in the cuticle is relatively small, they contribute to the majority of water sorption and hydrogen bonds^4,13^. Plant cuticle sorption curves have been found to be strongly influenced by the removal of polysaccharides, especially at high RH. The components of polar polysaccharides in tomato fruit cuticles are 25–33% cellulose, 26–30% pectin and 20–24% hemicellulose^8^. Polysaccharides are an important constituent of all cuticles studied to date and cellulose is present not only in leaf and fruit cuticles, especially on the inside surface, but also throughout the cuticle^8,21,30^. Cellulose has been visualised with gold labelling of poplar and pear cuticles and was observed throughout the whole cuticle.

**Fig. 1:**
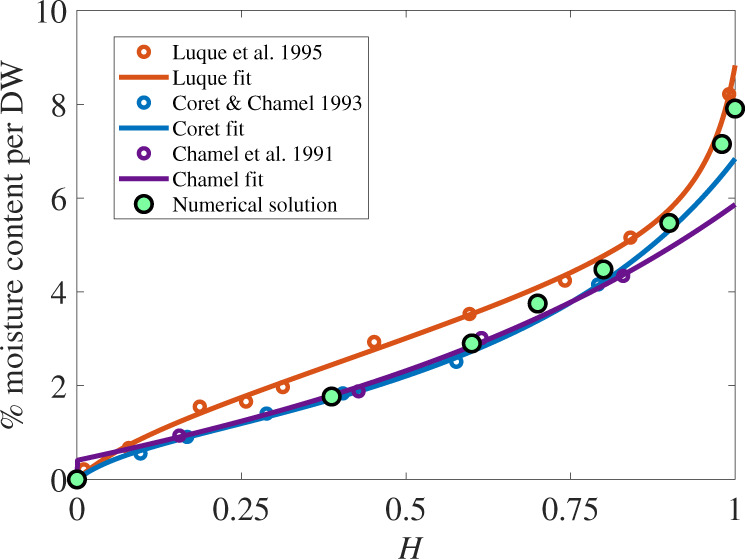
Validation plot compared to experimental data^4,10,12^ on isolated astomatous tomato fruit cuticles. The total experimental time in the data^4^ and model is 6 h for each point, and experimental data are shown as open circles. Relative humidity, *H*, is shown as a fraction on the *x*-axis and the percentage moisture content on the *y*-axis is calculated with Supplementary Eq. (8). The green dots are the numerical solution results of the cuticle model, and the solid curves are simple fits described around Supplementary Table 2.

The water sorption of isolated cuticles increases with RH^4^, with additional sorption at high humidity, as shown in Fig. 1 and Supplementary Fig. 1. When the isolated cuticle is treated to create a hydrolysed/cutin sample, which is free of polar polysaccharides, including crystalline cellulose, the increase at high humidities is much less pronounced and has a linear trend. The polar polysaccharides (cellulose) in the cuticle cause the large increase in sorption at high humidities. As there is more research around cellulose than hemicellulose and pectin in the cuticle, and it has been found to be the major polysaccharide^31^, we will henceforth refer to polar polysaccharides simply as cellulose.

Adsorption or binding of ionic compounds to the surfaces of isolated cuticles is possible, and the capacity of binding is more on the inside and less on the outside cuticle surface^32^. In some cases, the difference is 12–68 times, being higher on the inside surface. The timescale and amount of ions that penetrated varied depending on the application cuticle side for several plant species and compounds. Extending this mechanism to an intact leaf, the cuticle is more likely to gain ionic compounds from applied agrochemicals and aerosols deposited from the atmosphere and is less likely to lose ions from inside the plant leaf interior. Permeability to water has the same directional dependence as ionic compounds^33^. Cellulose was found in surfaces of cuticles in two independent works^33,34^, generally having a larger proportion on the inner surface. The presence of cellulose and directional dependence implies that water can bind to cellulose on the cuticle surface. When applying this to the whole leaf, water penetration entering from outside is more likely and the cuticle is less likely to lose water from the inside of the leaf. It is important to include a mechanism in a water sorption or penetration model where water is trapped or bound to the cuticle surface with some degree of asymmetry.

There is still the question of how and why this high sorption takes place at high humidity, as shown in Supplementary Fig. 1. Cellulose is highly hygroscopic and can sorb 30% of water per dry weight^35^, and polar polysaccharides, isolated from cuticles, can sorb even more at 49%^31^. Regarding polysaccharides in cuticles, very little work has been done on composition, molecular characteristics, physical or chemical behaviour^8^. Water molecules can adopt many interaction configurations of surrounding molecules due to the two hydrogen bond acceptor and donor sites. Water also provides many opportunities to be inserted into a polymer with the ability to play a multitude of roles^13^. Many studies using different experimental techniques^13,36–40^ have found three types of hydrogen bonds: cellulose to other cellulose groups, water to cellulose groups, and water to other water molecules^40^ (where water is attached to cellulose, noted to be tetrahedral in shape^36^). Above 80%RH, the share of bonds of water to other waters is the most abundant^36,40^. We surmise that at high humidity, above around 55%RH^31^, the cellulose to hydrogen bonds are mostly full, so water to water bonds form, and water sorption increases significantly at high humidity. We note that more research needs to be done, with particular focus on cellulose at high humidity and why water to water bonds form more frequently at high humidities than other kinds of bonds.

## Results

### Model framework

We describe a comprehensive mechanistic model for isolated cuticle water transport. The model takes the form of a nonlinear, one-dimensional diffusion model, including partial differential equations. We will briefly describe the modelling formulation that is based on the authors’ previous works^3,17,18^ and we refer the reader to these works for a full description of auxiliary Eqs. (5), (8) and (9) and Supplementary Eqs. (2) and (3).

Aqueous pores transport water and their properties vary between plant species, with RH, and are less influenced by temperature and the presence of waxes than the lipophilic pathway^6,27^. The sorption ability of cellulose is higher than other cuticle materials and it is not homogeneously distributed^30,33,34^. We model cellulose as a separate pathway from the aqueous pores, which is reasonable due to the results shown in Supplementary Fig. 1. We account for water that can travel in three pathways: aqueous pores (A), the lipophilic pathway (L), and cellulose (C). These three pathways are governed by different mechanisms, so must be modelled separately. Moisture concentration changes primarily along the cuticle membrane depth, *z* (0 ≤ *z* ≤ *b*). Moisture inside the cuticle is considered liquid water, which is a reasonable assumption based on experimental data^13,28^. This model will make additions to a simple diffusion model by accommodating the unique pathways of the cuticle. We incorporate the important governing mechanisms of swelling of the aqueous pores, climatic conditions such as temperature and RH that affect the pore swelling and adsorption, parameters that account for differences in plant species, including porosity and tortuosity, pore density and radius, cuticle thickness and binding to the cuticle surface due to cellulose. The model, as described in Eqs. (1)–(13), Supplementary Eqs. (2)–(8), parameters in Table 1 and constants in Supplementary Table 3, for the diffusion and adsorption of water into the three pathways of the cuticle, is as follows:

1 $$\left({\varepsilon }_{{{{\rm C}}}}+{\varepsilon }_{{{{\rm L}}}}\right)\,\frac{\partial c}{\partial t}+\frac{\partial \left({\varepsilon }_{{{{\rm D}}}}c\right)}{\partial t}=	 \left({\varepsilon }_{{{{\rm C}}}}\,{D}_{{{{\rm C}}}}+{\varepsilon }_{{{{\rm L}}}}\,{D}_{{{{\rm L}}}}\right)\ \frac{{\partial }^{2}c}{\partial {z}^{2}}+\frac{\partial }{\partial z}\left({D}_{{{{\rm A}}}}\frac{\partial ({\varepsilon }_{{{{\rm D}}}}c)}{\partial z}\right)\\ 	+S,\,\,0\, < \,z\, < \,b,\,t\, > \,0,$$

2 $$S=-\frac{2}{{r}_{{{{\rm A}}}}}\left(1-{\varepsilon }_{{{{\rm D}}}}\right)\frac{\partial {{{\Gamma }}}_{{{{\rm A}}}}}{\partial t}-{\rho }_{{{{\rm C}}}}\,\left(1-{\varepsilon }_{{{{\rm C}}}}\right)\frac{\partial {{{\Gamma }}}_{{{{\rm C}}}}}{\partial t},$$

where *c* is the concentration of liquid water in the cuticle, *ε*_D_, *ε*_C_ and *ε*_L_ are the porosities, *t* is time, *D*_A_, *D*_C_ and *D*_L_ are the diffusivities, *z* is the depth of the cuticle, *r*_A_ is the aqueous pore radius, Γ_A,C_ is the concentration of adsorbed water in the aqueous pores and cellulose, *S* is the adsorption term and *ρ*_C_ is the density of cellulose fibres. The subscripts A, D, C and L are aqueous pores (A is the entire aqueous pore and D is the aqueous pore available for diffusion), cellulose and lipophilic pathway. The parameters that change in space and time include *c*(*z*, *t*), *D*_A_(*z*, *t*), *r*_A_(*z*, *t*), *ε*_D_(*z*, *t*), Γ_A,C_(*z*, *t*), as described in Eqs. (5), (8), (9), (10), and (11).

**Table 1:** Model parameters.

Parameter	Definition	Value and units
*c*(*z*, *t*)	Concentration of water	mol/m^3^
*D*_A_(*z*, *t*), *D*_L_, *D*_C_	Diffusivity of water in the aqueous pores, lipophilic pathway and cellulose pathway	m^2^/s
*r*_A_(*z*, *t*)	Radius of aqueous pores	m
*t*	Time	s
*z*	Cuticle depth	m
*ε*_D_(*z*, *t*)	Aqueous pathway porosity for diffusion	
Γ_A_(*z*, *t*)	Concentration of water adsorbed per unit area in the aqueous pathway	mol/m^2^
Γ_C_(*z*, *t*)	Concentration of water adsorbed in cellulose	mol/kg
Δ*w*(*t*)	Weight increase of cuticle above dry weight	mg

The experimental set-up^4^, which is utilised to design the model including the domain (the cuticle) and initial conditions, consists of astomatous isolated tomato fruit cuticles, placed in a range of RHs. The cuticles are initially dry and the RH is increased in steps. The cuticles are weighed and the increase in weight, above the dry weight, is recorded in two different experiments, one at a near steady state at 6 h over a large range of humidities, and the other over 10 min and a small selection of humidities. Both experiments are at a constant temperature of 20 °C. Moisture enters the cuticle at both the outside and inside cuticle surfaces. At the boundaries (cuticle surfaces), water travels into the cuticle and adsorbs to the cuticle surfaces due to the presence of cellulose, where water is trapped and no longer available for diffusion, but can increase the cuticle weight. Water then enters the cuticle and can travel down the three pathways. In the aqueous pores, the free water molecules may travel through the cuticle via passive Fickian diffusion or be adsorbed in a monolayer to the aqueous pore walls, causing them to swell. In cellulose, free water travels through the cuticle via passive Fickian diffusion or adsorbs within the cuticle to cellulose fibres and on the cuticle surface. In the lipophilic pathway, water can diffuse through the cuticle and is influenced by temperature change. In Eq. (1), the last term is described in Eq. (2) of adsorption in the cuticle aqueous pores and cellulose. The term governing adsorption to aqueous pores in a monolayer is formulated by including the surface area of a pore over the pore volume including porosity, and the cellulose formulation instead utilises the density of cellulose with multilayer adsorption. Both free and adsorbed water increases the weight of the cuticle and are required to calculate Δ*w*, the weight increase of cuticle above dry weight, in Supplementary Eq. (7). The adsorption isotherms for Γ_A_ and Γ_C_ are described in more detail around Eqs. (9) and (10).

The boundary conditions for the cuticle surface mechanisms involved in sorption are significant. The cuticle experimental set-up is such that the RH is applied at both the outside and inside boundary conditions. The outside and inside cuticle surfaces, at *z* = 0 and *z* = *b*, are governed by the following boundary conditions:

3 $$	-{\left.\left[\left({\varepsilon }_{{{{\rm C}}}}{D}_{{{{\rm C}}}}+{\varepsilon }_{{{{\rm L}}}}{D}_{{{{\rm L}}}}\right)\frac{\partial c}{\partial z}+{D}_{{{{\rm A}}}}\frac{\partial ({\varepsilon }_{{{{\rm D}}}}c)}{\partial z}\right]\right|}_{z = 0}\\ =	-h\,\left({c}_{\infty }-c(0,t)\left(1-\frac{c(0,t)}{{c}_{{{{{\rm H}}}}_{2}{{{\rm O}}}}^{{{{\rm pure}}}\,}}\right)\right)-{k}_{1}\,c(0,t),\quad t \; > \; 0,$$

4 $$	-{\left.\left[\left({\varepsilon }_{{{{\rm C}}}}{D}_{{{{\rm C}}}}+{\varepsilon }_{{{{\rm L}}}}{D}_{{{{\rm L}}}}\right)\frac{\partial c}{\partial z}+{D}_{{{{\rm A}}}}\frac{\partial ({\varepsilon }_{{{{\rm D}}}}c)}{\partial z}\right]\right|}_{z = b}\\ =	h\,\left({c}_{\infty }-c(b,t)\left(1-\frac{c(b,t)}{{c}_{{{{{\rm H}}}}_{2}{{{\rm O}}}}^{{{{\rm pure}}}\,}}\right)\right)-{k}_{2}\,c(b,t),\quad t \; > \; 0,$$

where *h* is the moisture transfer coefficient for moisture going into the cuticle, *c*_*∞*_ is the atmospheric vapour concentration of air far from cuticle as a function of temperature, ${c}_{{{{{\rm H}}}}_{2}{{{\rm O}}}}^{{{{\rm pure}}}\,}$ is the concentration of water as a function of temperature and *k*_1_ and *k*_2_ are the rate constants for water binding to cellulose on the outside and inside cuticle surfaces. Equations (3) and (4) are Robin-type boundary conditions. Equations (3) and (4) are analogous to Newton’s law of heating and show that the flux of moisture across the boundary is proportional to the difference between the atmospheric vapour concentration and moisture levels on the surface of the cuticle. We have included a logistic growth formulation that prevents the concentration of water exceeding that of pure water, necessary due to the values of the constants in the equations. Water can adsorb to the cellulose in the cuticle^32–34^ and the final term of Eqs. (3) and (4) describes the adsorption to cellulose on the cuticle surface. These terms show that adsorption to the surface increases proportional to the surface moisture concentration. The parameters *k*_1_ and *k*_2_, the rate constants for water adsorbing to cellulose on the cuticle surfaces, are functions of RH and the amount of cellulose present, as described in Supplementary Eq. (5) and Supplementary Table 3. The outer and inner surfaces are similar, except that they contain different amounts of cellulose, so different amounts of water can bind, as described in Supplementary Eq. (5) and Supplementary Table 3.

### Model validation

The model, as described in Eqs. (1)–(13) and Supplementary Eqs. (2)–(8), is solved numerically. We then validate the model solution against experimental data^4^. We validate the model over two sets of results, one with a range of RHs, and the second with a selection of humidities and time. Figure 1 shows the results of the model (green dots), compared to the experimental data, over a range of RHs. Due to the variable nature of cuticles, we have compared the model results to three experiments on isolated tomato fruit cuticles with a similar experimental set-up (open circles)^4,10,12^ and fitted (continuous curves) their data with a simple equation, as described in Supplementary Table 2. In Fig. 1, we can see the model compares well to all three experiments over the range of humidities. The Luque et al.^12^ data set is included as it includes high humidities, but the model is based on the Chamel et al.^4^ experimental set-up, so over all humidities the model compares well to Chamel et al. The model increases to a large degree (above a linear trend), at very high humidities, at 90%RH and 99%RH, which is the desired result. This trend at high humidities is largely the result of water attaching to the cellulose on the outside and inside cuticle surfaces, discussed later (see below).

**Fig. 2:**
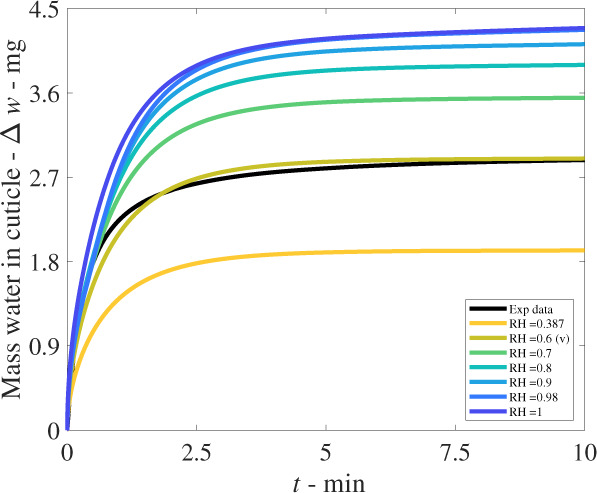
Validation plot compared to experimental data (Exp data in black) at 60%RH. The 60%RH cuticle model solution (green brown) can be seen corresponding closely to the experiment in black. (v) represents the solution used for validation. The total experimental time here is 10 minutes. We reproduce the experimental data, as described around Supplementary Eq. (1).

**Fig. 3:**
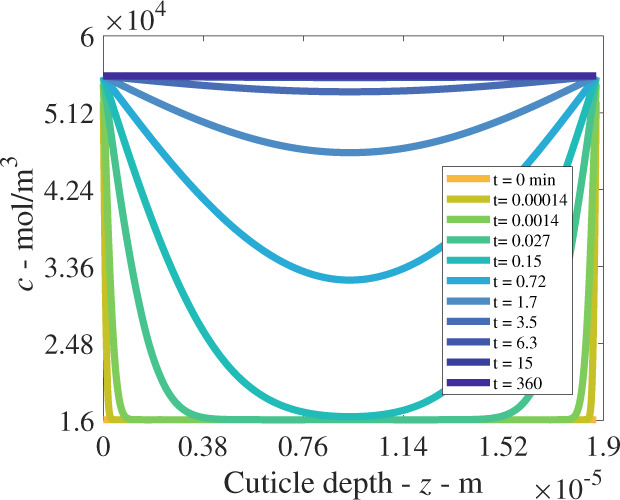
Plant cuticle model results at a single relative humidity of 60%RH, for the concentration of diffusing water molecules over 6 h. Over the depth of the cuticle, results are shown at a selection of times. The initial condition is shown in orange and the boundary conditions for the outside and inside cuticle surfaces are located at cuticle depth 0 and 1.87 × 10^−5^ m, respectively.

In Fig. 2, we see the model compares well to the experimental data, at 60%RH over time, and the change in dry weight increases proportional to RH. The initial rapid increase is largely produced by cellulose and aqueous pores, with less influence from the lipophilic pathway, discussed later. In Fig. 3, we see the concentration plot of water molecules at a selection of times with the depth within the cuticle. Initially, (orange) the water concentration is low and constant through the cuticle. Then, as time increases, the concentration at the boundaries increases and then gradually diffuses into the cuticle. At late times, the cuticle is close to the concentration of pure water. We note Fig. 3 alone shows diffusing water molecules and not adsorbing molecules. Due to the asymmetric boundary conditions, the concentration of water at the two boundaries is similar but not identical.

**Fig. 4:**
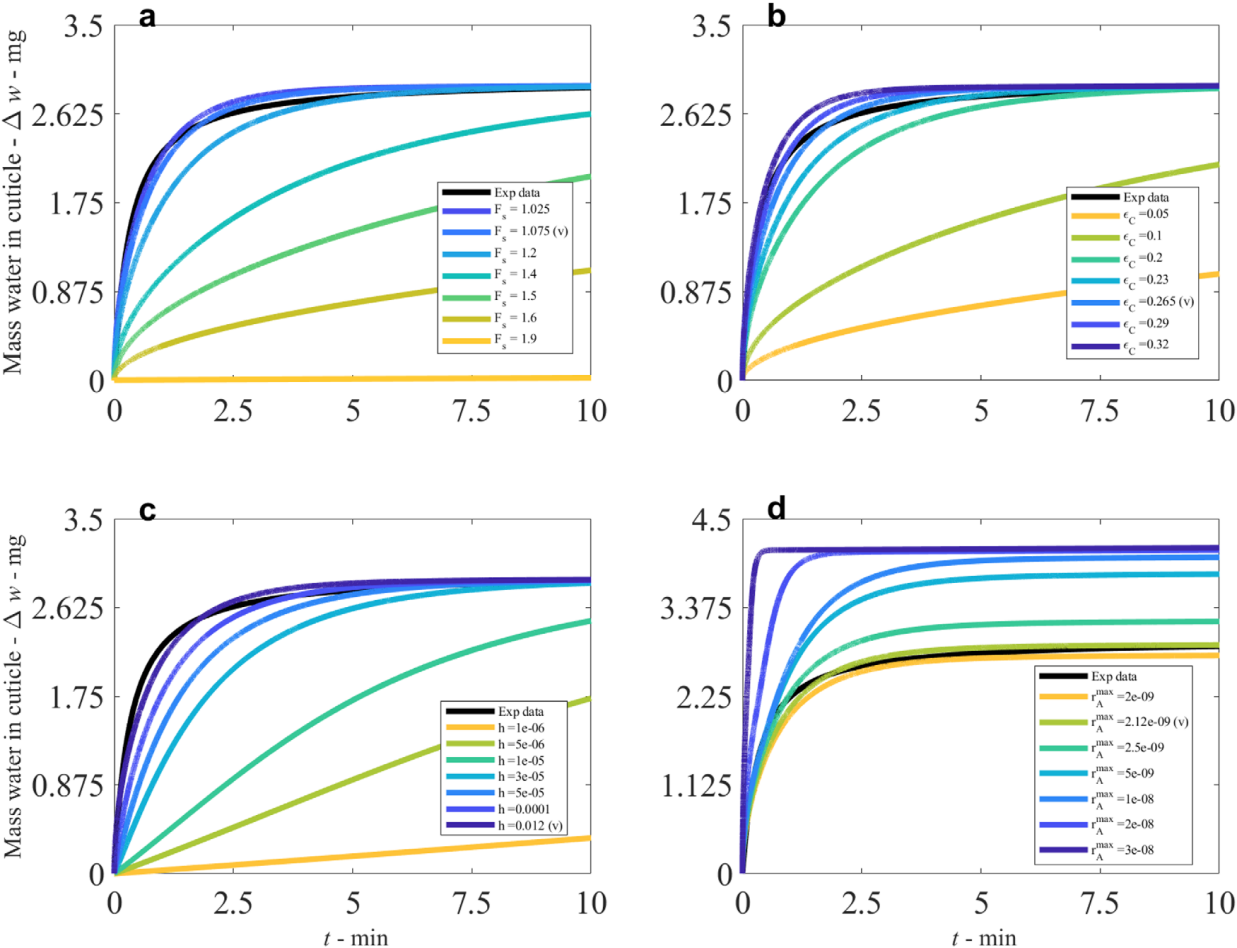
Sensitivity analysis of the model, compared to the results for validation (v) and the experimental data (Exp data in black) as a reference point, all performed at 60%RH. All model parameters are kept constant except the sensitivity parameter. **a** shows the sensitivity of *F*_s_, the fractal scaling dimension, analogous to the tortuosity, **b** shows *ε*_C_, which is the porosity of cellulose, **c** shows *h*, the moisture transfer coefficient at the boundaries, and **d** shows ${r}_{\,{{{\rm A}}}}^{{{{\rm max}}}\,}$, the maximum radius of aqueous pores. Note for all the subfigures, the relationship is directly proportional, except for *F*_s_, which has an inverse relationship.

We conduct a sensitivity analysis of the model, based on the validation result (v) in Fig. 2 at 60%RH, keeping all the parameters the same, then utilising the one-factor-at-a-time method to determine the model parameter sensitivities, as shown in Fig. 4. A selection of sensitivities is discussed below. Figures 1 and 2 have already displayed the sensitivity of RH and been validated with the experimental data. In Fig. 4a, we see the results of changing *F*_s_, the fractal scaling dimension, analogous to the tortuosity, of the three pathways. The increase in weight of the cuticle is very sensitive to the tortuosity of the pathways, as this modifies the diffusivities. This is the only inverse relationship produced, with increasing *F*_s_ meaning a more tortuous and longer pathway that is more difficult to cross, slowing, and limiting the diffusivity and diffusion of the three pathways. When applying this result to isolated cuticle experiments, the tortuosity of aqueous pores will differ significantly between plant species cuticles, due to plant anatomy such as lamellate structures, thickness, orientation of pores and plant age^5,21,41^. Therefore, the significant effect of *F*_s_ indicates that the variation of plant species itself has a significant effect on water transport, and this is seen experimentally^5^.

In Fig. 4b, the sensitivity of *ε*_C_ is shown, the porosity of cellulose. This parameter has a strong direct relationship to mass increase and shows similar (but direct not inverse) trends to *F*_s_, as these parameters modify the effective diffusivity of cellulose (Eq. (6)). A larger porosity value implies more pores for diffusion, so this trend is as expected. The strong effect of modifying a parameter that only applies to this one pathway implies cellulose contributes significantly to mass increase of the cuticle. In Fig. 4c, we see the sensitivity to *h*, the moisture transfer coefficient at the boundaries. The parameter *h* has a large effect as it modifies the three pathways and changes the time when the water enters the cuticle but not the maximum value at late times. Figure 4d shows ${r}_{\,{{{\rm A}}}}^{{{{\rm max}}}\,}$, the maximum radius of the aqueous pores. A direct relationship exists, and increasing the maximum pore size promotes faster diffusion and adsorption into the pores. The maximum aqueous pore radius has a somewhat significant effect over the change in mass increase of the cuticle. The implication for this effect of changing this parameter for aqueous pores is that aqueous pores do contribute somewhat to the overall mass increase, but significant contributions also are produced by cellulose porosity, observed by comparing the change in mass between the maximum and minimum values in Fig. 4b, d.

Additional parameters studied include the rate coefficient for cellulose binding on the boundaries, *k*, the porosity of the lipophilic pathway, *ε*_L_, and temperature, *T*. In Supplementary Figs. 2 and 3, the rate constant for binding at the boundary conditions due to cellulose, *k*, has very little effect on mass increase in the short term but a large effect over longer times. This indicates that binding is over a long timescale and too slow compared to the diffusion timescale, and that cellulose binding contributes significantly over long periods to the results shown in Fig. 1. Two timescales are evident here, a short diffusion and adsorption timescale governed largely by cellulose and aqueous pores diffusion and adsorption inside the cuticle, and a longer timescale governed by adsorption onto the cellulose at the boundaries, and these two timescales are reflected by both experiments and mathematical models^3,4,18^. The mechanism of binding on the cuticle surface by cellulose is crucial to include in a water sorption model so we can validate the results in Fig. 1, and must be included, as it was in previous cuticle models^3,17,18^. The importance of surface binding of water by cellulose has also been found in the well-established literature^35^. In Supplementary Fig. 4, the porosity of the lipophilic pathway, *ε*_L_, has a weak effect on mass increase, which implies the lipophilic pathway does contribute to mass increase somewhat, but not to a significant degree in this study. In Supplementary Fig. 1, wax extraction only had a small effect^4^ and results found elsewhere^5^ agree where water travels mostly down the aqueous pathway and less down the lipophilic pathway. We note that some studies^10,11^ have used other techniques, chemicals, pre-treatments and adjuvants, which may increase the role of the lipophilic pathway for water penetration. In Supplementary Fig. 5, there is a small effect of changing temperature, *T*, in the model, where the lipophilic pathway is influenced by *T*, as shown in Supplementary Table 3. The lipophilic pathway is influenced by temperature and aqueous pores less so^6,27^ and Chamel et al.^4^ find only a weak relationship between temperature and mass increase of the cuticles studied. Therefore, these results are in keeping with the literature^4,6,27^. If the lipophilic pathway role was increased, the lipophilic model constants could be altered and small modifications can be made to the model, as outlined elsewhere^18^.

The parameters that are highly influential over sorption of the cuticle are related to variations in plant species, including ${F}_{{{{{{\rm{s}}}}}}},\;b,\;{r}_{\,{{{\rm A}}}}^{{{{\rm max}}}}$ and *η*_A_. The parameters related to cellulose, including *ε*_C_, *k*, and the parameters related to *k* and Γ_C_, are also highly influential over sorption in the model, and this aligns with experimental data, as cellulose sorbs significant amounts of water in cuticles^4,31^. RH also has a large effect on mass increase. Our results from the sensitivity analysis are feasible. Considering all the results from the sensitivity analysis, we find that cellulose and the aqueous pores contribute to mass increase of the cuticle in the shorter timescale (10 min), while the lipophilic pathway contributes to a small degree. In the longer timescale (6 h), diffusion in cellulose and binding to the cellulose at the boundaries drives the mass increase.

## Discussion

The model can simulate water sorption in the cuticle with any RH, temperature, content of cellulose on the surface, cuticle thickness, aqueous pore radius and pore density. The model can be theoretically applied to any plant species by modifying the constants $b,\;{{{{{\rm{DW}}}}}},\;{c}_{{{{\rm min}}}},\;{F}_{{{{{{{\rm{s}}}}}}}},\;n,\;{r}_{\,{{{\rm A}}}}^{{{{\rm max}}}},\;{\rho }_{{{{\rm C}}}},\;{{{{{\rm{and}}}}}}\;{\eta }_{{{{\rm A}}}}$. The model does not include any mechanisms that are specific to any one plant species, and future work could include validating the results for other plant species, noting that the trends for other plant species are very similar in Chamel et al.^4^. The model only requires one set of data to be trained on, and many parameters are available in the literature, as described in Supplementary Table 3.

When extending this model to an attached plant leaf (though not considering stomatal interactions), the previously held assumption was that the interior was 100% humidity or equivalent to liquid water. With these conditions, water loss to the environment may occur through the cuticle from the interior of the plant, when the environment is at low RHs. For transport in the other direction (inwards), new data (S. C. Wong and G. D. Farquhar, unpublished data) suggests that the humidity at the cuticle interior is <100%RH in some conditions, and this could assist with the modelling, when this work is expanded to the attached plant leaf. Considering transport in astomatous cuticles attached to leaves, at isothermal conditions, if the interior boundary condition or initial condition of the cuticle is <100%RH, water will enter the cuticle and subsequently the plant from the exterior, as water goes down the concentration gradient based on passive diffusion.

When extending this model to consider cuticle transpiration, it is difficult to extrapolate the results, especially when stomata are involved^9^ and high temperatures^42^. Transpiration is beyond the scope of this work as additional experimentation is required. The mechanisms may be much more complicated than isolated astomatous cuticle water sorption as the cuticle is attached to the leaf, which may also be attached to the plant.

With further experimental research, the following could be investigated and potentially incorporated into a model, including how pectin, hemicellulose^8^ and xylan adsorption and diffusion differs from cellulose, how the structure of cellulose within the cuticle influences adsorption and diffusion and the influence of phenolics including flavonoids on water sorption, which may relate to their influence on plant cuticle biomechanics^43^ and be especially relevant at different growth stages.

The mechanism of water adsorption and transport in cellulose could lead to a further understanding of the transport of hydrophilic uncharged agrochemicals though cuticle. Uncharged methyl glucose has been found to have influencing mechanisms that differed from hydrophilic ionic or lipophilic compounds, indicating an alternative hydrophilic pathway within the cuticle. In all, 60% of methyl glucose diffused across the aqueous pathway, while 40% used an alternative pathway^6,44^. Perhaps this other pathway could involve cellulose. We note that if the isolated cuticle was being heated and subsequently drying out, there will be vapour present inside the cuticle, as was found experimentally^13^. To model the transport of vapour and liquid in a heated cuticle, the model could be adapted, as shown elsewhere for other plant materials^45^.

Describing the aqueous pathway as a pore is one way to represent this pathway^6,46^. This is a suitable way to include these pathways in a mathematical model with a continuous modelling approach, where these pores or voids may be very small or grow larger and fill with water and change size in space and time with water content. We note that this pathway has also been described as a dynamic aqueous continuum^47,48^, that is, only continuous through the cuticle when polar functional groups are clustered^6^. Here we develop this further by separately modelling aqueous pores and cellulose, along with the lipophilic pathway, totalling three pathways, which is a more comprehensive approach. Modelling three separate pathways is important as their governing mechanisms are different. With further mechanistic research, a more defined view of the aqueous continuum can develop to further understand water transport in the cuticle and leaf. We note some research has been carried out on the cuticle and cellulose, but more research is needed for comprehensive insights into water transport in the cuticle, and this is a key reason for employing mathematical modelling here.

To conclude, we describe a comprehensive model for isolated cuticle water transport to move towards a better understanding of the transport of water within an attached intact leaf. This is the first comprehensive mechanistic model to simulate and validate moisture transport in cuticles. We bring together both new and old knowledge and create a more mechanistic perspective of water transport in the plant cuticle. This paper has highlighted the importance of including mechanisms influencing water transport in cellulose in a cuticle model. The model validates well and the sensitivities align with the well-established literature. By understanding the governing mechanisms, we can move towards improved modelling of whole attached plant leaves, agrochemical formulation development and application, understanding water loss from plants in a time of changing climate conditions and transport of liquid and gaseous water applied to the cuticle.

## Methods

### Model description

The diffusivities for water travelling in the three pathways are as follows, where the formulations are similar and the lipophilic pathway is governed by temperature:

5 $${D}_{{{{\rm A}}}}(z,t)={D}_{{{{{\rm H}}}}_{2}{{{\rm O}}}}^{{{{\rm bulk}}}\,}\ {{\varepsilon }_{{{{\rm D}}}}}^{\left(\frac{{F}_{{{{\rm s}}}}}{2-{F}_{{{{\rm s}}}}}\right)},\quad 0 \; < \; z \; < \; b,\,t \; > \; 0,$$

6 $${D}_{{{{\rm C}}}}={D}_{{{{{\rm H}}}}_{2}{{{\rm O}}}}^{{{{\rm bulk}}}\,}\ {{\varepsilon }_{{{{\rm C}}}}}^{\left(\frac{{F}_{{{{\rm s}}}}}{2-{F}_{{{{\rm s}}}}}\right)},$$

7 $${D}_{{{{\rm L}}}}={D}_{{{{{\rm H}}}}_{2}{{{\rm O}}}}^{{{{\rm bulk}}}\,}\ {e}^{\left(\frac{-E}{RT}\right)}\ {{\varepsilon }_{{{{\rm L}}}}}^{\left(\frac{{F}_{{{{\rm s}}}}}{2-{F}_{{{{\rm s}}}}}\right)},$$

where ${D}_{{{{{\rm H}}}}_{2}{{{\rm O}}}}^{{{{\rm bulk}}}\,}$ is the self/bulk diffusion coefficient of water as a function of temperature, *F*_s_ is the fractal scaling dimension, analogous to tortuosity, and *E*, *R* and *T* are constants described in Supplementary Table 3. The formulation for the changing aqueous pore radius is as follows, with more details provided elsewhere^17^:

8 $${r}_{{{{\rm A}}}}(z,t)={r}_{{{{{\rm H}}}}_{2}{{{\rm O}}}}\left(1+{\left(\sin \left({\left({{{\Gamma }}}_{{{{\rm A}}}}{r}_{{{{{\rm H}}}}_{2}{{{\rm O}}}}^{2}N\right)}^{-1}\right)\right)}^{-1}\right),\quad 0 \; < \; z \; < \; b,\,t \; > \; 0.$$

The following equations are utilised to model adsorption:

9 $${{{\Gamma }}}_{{{{\rm A}}}}(z,t)=\frac{{{{\Gamma }}}_{{{{\rm S}}}}\,\beta \,c}{1+\beta \,c},\quad \quad \quad \quad 0 \; < \; z \; < \; b,\,t \; > \; 0,$$

10 $${{{\Gamma }}}_{{{{\rm C}}}}(z,t)=\frac{{{{\Gamma }}}_{{{{\rm SC}}}}\,{\beta }_{{{{\rm C}}}}\,K\,c}{\left({c}_{{{{{\rm H}}}}_{2}{{{\rm O}}}}^{{{{\rm pure}}}\,}-K\,c\right)\left(1+K\,\left({\beta }_{{{{\rm C}}}}-1\right)\,\frac{c}{{c}_{{{{{\rm H}}}}_{2}{{{\rm O}}}}^{{{{\rm pure}}}\,}}\right)},\quad \quad 0 \; < \; z \; < \; b,\,t \; > \; 0,$$

where Γ_A_ is the concentration of water adsorbed per unit area in aqueous pores, Γ_C_ is the concentration of water adsorbed in cellulose and the constants Γ_S_, *β*, Γ_SC_, *β*_C_, *K* and ${c}_{{{{{\rm H}}}}_{2}{{{\rm O}}}}^{{{{\rm pure}}}\,}$ are described in Supplementary Table 3. Cellulose adsorption is modelled with a water adsorption isotherm model, which will contribute significantly at high humidity, due to the formation of multilayers of water. The adsorption for cellulose is modelled with the Guggenheim, Anderson, and De Böer (GAB) isotherm (based on the Brunauer–Emmett–Teller isotherm), Γ_C_, as shown in Eq. (10)^49–52^. The GAB isotherm describes water adsorption as a monolayer, which can then form multilayer at high humidities. Water adsorption to the aqueous pores is modelled with a Langmuir isotherm, as shown in Eq. (9), which describes adsorption as a monolayer and further details are discussed in previous works^17^. The radius for the aqueous pore for diffusion of water molecules is slightly smaller than the entire pore (*r*_A_), and water molecules are arranged in a monolayer or closed Steiner chain on the pore surface. We account for this by formulating porosity as follows:

11 $${\varepsilon }_{{{{\rm D}}}}(z,t)=\pi {\left(\left({r}_{{{{\rm A}}}}-2{r}_{{{{{\rm H}}}}_{2}{{{\rm O}}}}\right)\left(\sqrt{{\eta }_{{{{\rm A}}}}}+\frac{1}{L}\right)\right)}^{2},\,0\; < \; z \; < \; b,\,t \; > \; 0,$$

where *ε*_D_ is the porosity of the aqueous pores for diffusion, ${r}_{{{{{\rm H}}}}_{2}{{{\rm O}}}}$ is the Van der Waals radius of a water molecule, *η*_A_ is the density of aqueous pores in cuticle and *L* is the control volume length. The term ${r}_{{{{\rm A}}}}-2\,{r}_{{{{{\rm H}}}}_{2}{{{\rm O}}}}$ accounts for the smaller pore radius, and more details are available elsewhere on the formulation^17^. The initial conditions of the model dictate a dry cuticle that has a small amount of water present and the smallest aqueous pore radius where free water can exist is described as follows:

12 $$c(z,0)={c}_{{{{\rm min}}}},\quad 0\le z\le b,$$

13 $${r}_{{{{\rm A}}}}(z,0)=3\,{r}_{{{{{\rm H}}}}_{2}{{{\rm O}}}},\quad 0 \; < \; z \; < \; b,$$

where *c*_min_ is the concentration of water in a somewhat dry cuticle as a function of RH and a radius of three waters is the minimum to allow the transport of water molecules.

The model, as described in Eqs. (1)–(13) and Supplementary Eqs. (2)–(8), is solved numerically, similar to previous models^3,18^. We use ‘ode15i’ within MATLAB^®^, with a finite volume method, averaging of the diffusivity function, *D*_A_(*z*, *t*), for aqueous pores at the control volume faces, and discretise the model’s partial differential equations with second-order central differences to approximate the spatial derivatives, with evenly distributed nodes. The fitted parameters as described in Supplementary Table 3 are found by fitting to the data^4^ at 60%RH, then keeping all the parameters the same for all other simulations and only changing the humidity to match the experimental humidity.

The timescale of moisture sorption is important as it informs the development of models. Sorption from 5 plant species starts to level out at around 5–10 min at humidities <70% but high humidities are not measured, and samples are also measured for 6 h minimum but the maximum is not stated at each humidity and this data set is not measured with time^4^. For moisture sorption in cellulose, 20%RH took around 1 h, while sorption at 95%RH took 5.6 h to level out^39^. An experimental time of 2–3 days is common with gravimetric techniques (Tredenick, E. C., Stuart-Williams, H. & Enge, G., 2021, Materials on plant leaf surfaces are deliquescent in a variety of environments, unpublished) and agrochemical penetration experiments typically occur over 2–3 days^17^, while with a radioactive water sorption technique in cuticles, 20–28 h was used^28^. Therefore, we can conclude that, for the Chamel et al.^4^ data after 6 h, the sorption has not necessarily reached equilibrium in a mathematical sense, and data measured with time over 3 days is necessary to establish the true point of equilibrium. We note it is important to define the usage of the term equilibrium, and here we define it as the time at which the rate of change or time derivative is zero. However, in an experimental sense, the experiment will often end sooner at a near steady state, due to a variety of reasons. The timescale of aqueous pore swelling is also an important consideration, but there has not yet been a definitive study with a range of RHs and plant species to define this timescale and more work needs to be done. Here aqueous pores swell close to their maximum at 9 min but may take longer to reach equilibrium.

## Supplementary information

Supplementary Information

## Data Availability

The experimental data that the model was validated against are available in Chamel et al.^4^.

## Code availability

The simulation and post-processing codes that have been used to produce the results of this study are available from the corresponding author upon reasonable request.
