# Publisher Correction: Dynamics of moisture diffusion and adsorption in plant cuticles including the role of cellulose

**DOI:** 10.1038/s41467-021-25776-0

**Published:** 2021-09-30

**Authors:** E. C. Tredenick, G. D. Farquhar

**Affiliations:** ARC Centre of Excellence in Translational Photosynthesis, Division of Plant Science, Research School of Biology, The Australian National University, Canberra, ACT Australia

**Keywords:** Permeation and transport, Plant physiology, Applied mathematics

Correction to: *Nature Communications* 10.1038/s41467-021-25225-y, published online 19 August 2021.

The original version of this Article contained an error in Equation 10. The term at the end of the denominator was presented as a product rather than a fraction, and incorrectly read:

$${\Gamma }_{{{{{{\rm{C}}}}}}}(z,t)=\frac{{\Gamma }_{{{{{{\rm{SC}}}}}}}\,{\beta }_{{{{{{\rm{C}}}}}}}\,K\,c}{({c}_{{{{{{{\rm{H}}}}}}}_{2}{{{{{\rm{O}}}}}}}^{{{{{{\rm{pure}}}}}}}-K\,c)(1+K\,({\beta }_{{{{{{\rm{C}}}}}}}-1)\,c{c}_{{{{{{{\rm{H}}}}}}}_{2}{{{{{\rm{O}}}}}}}^{{{{{{\rm{pure}}}}}}})},\,0 \, < \, z \, < \, b,\,t \, > \, 0,$$

The correct form of Equation 10 is:

$${{{\Gamma }}}_{{{{\rm C}}}}(z,t)=\frac{{{{\Gamma }}}_{{{{\rm SC}}}}\,{\beta }_{{{{\rm C}}}}\,K\,c}{\left({c}_{{{{{\rm H}}}}_{2}{{{\rm O}}}}^{{{{\rm pure}}}\,}-K\,c\right)\left(1+K\,\left({\beta }_{{{{\rm C}}}}-1\right)\,\frac{c}{{c}_{{{{{\rm H}}}}_{2}{{{\rm O}}}}^{{{{\rm pure}}}\,}}\right)},\quad \quad 0 \; < \; z \; < \; b,\,t \; > \; 0,$$

This has been corrected in both the PDF and HTML versions of the Article.

