# Supplementary material for: Dynamics of moisture diffusion and adsorption in plant cuticles including the role of cellulose
Source: Nat Commun. 2021 Aug 19;12:5042. doi: 10.1038/s41467-021-25225-y (PMC8377085; doi:10.1038/s41467-021-25225-y)
Supplement: Supplementary file 1 — Supplementary Information [file 41467_2021_25225_MOESM1_ESM.pdf]

Supplementary Material for

Dynamics of Moisture Diffusion and Adsorption in Plant Cuticles Including the Role of Cellulose

E. C. Tredenick [1,*] and G. D. Farquhar [1]

[1]ARC Centre of Excellence in Translational Photosynthesis, Division of Plant Science, Research School of Biology, The Australian National University, Canberra, ACT 2601, Australia.

*e-mail: eloise.tredenick@anu.edu.au

Supplementary Note 1: Experimental Data Fitting

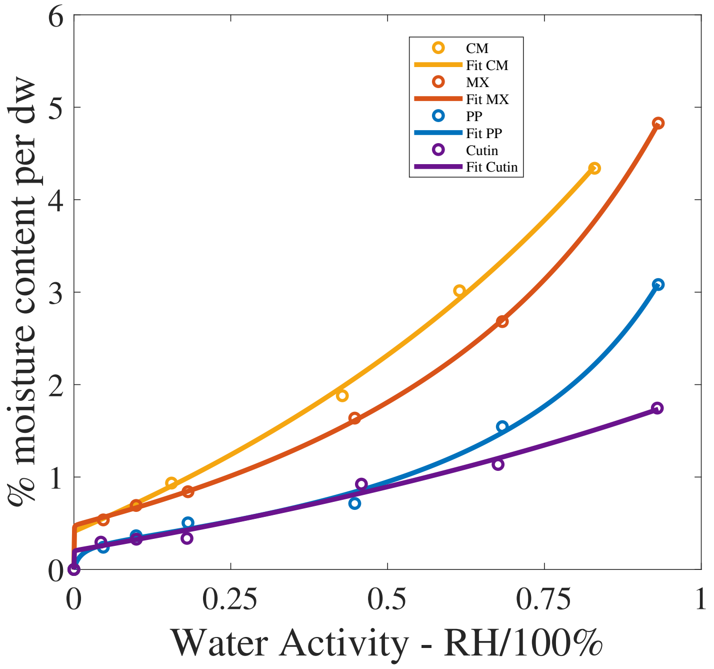

Supplementary Figure 1: Moisture sorption curves for the cuticle membrane (CM), polymer matrix (MX) membranes, cutin (free of polysaccharides), and polar polysaccharides (PP) only, which is MX minus cutin. The MX and CM curves are very similar. When PPs are removed (referred to as cutin) in purple, the large nonlinear increase in sorption at high humidities is no longer present and the sorption increases linearly. The large nonlinear increase in the PP curve can clearly be seen at high humidities, indicating PPs are the reason for this trend. We fitted[1] the data[2] using $y = a\,b\,x/(1+a\,x)+c\,d\,x/(1-c\,x)$, where y is the percentage moisture adsorption per dry weight and x is the water activity or RH/100%, with parameters shown in Supplementary Table 1.

Supplementary Table 1: Fit parameters for Supplementary Figure 1.

Parameter	CM	MX	PP	Cutin
a	1.44×10^5	1.8×10^4	91.64	2.04×10^4
b	0.41	0.48	0.28	0.2
c	0.46	0.67	0.78	0.31
d	6.36	2.67	1.06	3.75
R^2 %	99.9	99.9	99.6	99.0

The experimental data over a time of 10 minutes, as shown in Fig. 2, was shown in the original text[2] as uncorrected for water sorption by the vessel or basket holding the sample that is also being weighted and can also adsorb water. We have corrected for the vessel here by fitting two Langmuir isotherms (see equation (9)), then the sample minus the vessel, to produce the weight of water in the cuticle, $\Delta w(t)$, at 57%RH, with an R-squared value better than 99%, as follows:

$$\Delta w(t) = \frac{3.758\times3.437\times t}{3.437\times t+1} - \frac{6.056\times0.7808\times t}{6.056\times t+1}, \quad (1)$$

where t here is time in minutes. The original work[2] did not provide error bars, and the two sets of data points, for the sample data not corrected for by the vessel and the data for the vessel, were not conducted at matching relative humidities, hence we are only able to produce a fitted curve, not individual data points.

Supplementary Table 2: We find fitted parameters for solid lines in Fig. 1, using $y = a\, b\, H/(1 + a\, H) + c\, d\, H/(1 - c\, H) + e\, H$, where y is the percentage moisture adsorption per dry weight[1] and H is the water activity or RH/100%.

Parameter	Luque[1]	Coret and Chamel[3]	Chamel[2]
a	8.55	10.76	3.89×10^4
b	0.99	0.79	0.41
c	0.95	0.66	0.46
d	0.22	3.09	6.35
e	4.02	0	0
R^2 %	99.83	99.82	99.85

Supplementary Note 2: Additional Model Description

Here we describe additional equations, included in the model, as shown in Supplementary Equations (2)–(8) and the modelling constants in Supplementary Table 3. The parameters Γ_S and β are calculated as follows:

$$\Gamma_S = \left(r_{H_2O}^2\, N \arcsin\, \gamma\right)^{-1}, \tag{2}$$

$$\beta = \frac{\arcsin\, \gamma}{c_{H_2O}^{pure}\left(\arcsin\left(\dfrac{r_{H_2O}}{r_A^{max}\, H^2 - r_{H_2O}}\right) - \arcsin\, \gamma\right)}, \tag{3}$$

$$\gamma = \frac{r_{H_2O}}{r_A^{max}\, H - r_{H_2O}}. \tag{4}$$

The constant Γ_S, described in Supplementary Equation (2), is described elsewhere,[4] γ is defined in Supplementary Equation (4), and here the maximum pore radius, r_A^{max}, is limited by relative humidity, H. The constant β, as described in Supplementary Equation (3), is formulated utilising equation (9) and Supplementary Equation (2), simplifying and rearranging. To calculate the binding of water to cellulose, k_2, as described in Supplementary Table 3, on the surface of the cuticle as a function of humidity, H, we utilise the following equation to find W_C,

$$W_C = K_1\left(\frac{K_2\, H}{1 + K_2\, H} + \frac{K_3\, H}{1 - K_3\, H}\right), \tag{5}$$

where W_C is the weight of water adsorbed per gram of dry solid as a fraction, H is the relative humidity as a fraction or water activity, K_1 is the number of strong binding sites and equal to 0.05, K_2 is the attraction of these sites and equal to 7.43, and K_3 is related to the water activity of the solid and equal to 0.907.[5] All parameters are dimensionless and a_{surf} scales the outside surface, as there is less cellulose[6,7] on the outside surface (see Supplementary Table 3).

Supplementary Note 3: Conversion of Concentration to Weight

To convert the final solution from a concentration to a weight in mg, including the adsorbed water, the following equations are applied. The initial condition is removed from the solution, as the initial condition is equivalent to the dry weight, to produce

$$c^w(z,t) = c(z,t) - c_{min}, \tag{6}$$

where c^w is the concentration of water without the initial condition, c is the concentration of free water and solution to the model, and c_{min} is the initial condition.

The experimental data are given as the difference between the wet weight, at a given relative humidity, and dry weight over time; therefore to convert the concentration to the total weight in mg, the following equation is utilised. The first integral is considered over space (resulting in a solution at each point in time), and the second is a cumulative integral over time, and the first three terms are ions in the cuticle, adsorbed in aqueous pores and adsorbed in cellulose, and the cumulative integral term is the ions adsorbed to cellulose at the two cuticle surfaces,

$$\Delta w(t) = M_w\, f\, n\left[\int_0^b \left[A_{CM}\, c^w(z,t) + 2\,\pi\, r_A(z,t)\, \Gamma_A(c^w(z,t)) + \frac{f\, A_{CM}\, \rho_C\, \Gamma_C(c^w(z,t))}{\Gamma_{SC}\, M_w}\right] dz + A_{CM}\int_0^{t_{final}} \left[k_1\, c^w(0,t) + k_2\, c^w(b,t)\right] dt\right], \tag{7}$$

where $\Delta w(t)$ is the weight increase over dry weight in mg, M_w is the molecular weight of water, f is the value 10^3 and converts g to mg or kg to g, n is the total number of cuticle discs used in the experiment, t_{final} is the final experimental time and is 10 minutes here, A_{CM} is the area of one cuticle disc, z is the thickness of the cuticle, Γ_A is the adsorption of water to the aqueous pores, Γ_C is the adsorption of water to cellulose, Γ_{SC} is the saturated concentration of water adsorbed in cellulose, r_A is the aqueous pore radius, t is time and k_1 and k_2 are the rate constants for binding to cellulose on the cuticle surfaces. The formulation of the area for the moles adsorbed per aqueous pore, using Γ_A, is based on the circumference of the aqueous pore that is circular in cross-section.

To convert Δw to a percentage increase over the dry weight at the end time, we utilise the following:

$$\%\text{moisture content per DW} = \frac{\Delta w(t_{\text{final}})\ 100\%}{DW\ n}, \tag{8}$$

where %moisture content per DW is the percentage gain of water content over the dry weight, DW is the total dry weight and t_{final} is 6 hours here. The resultant change in weight, $\Delta w(t)$, is a vector and can be seen in Fig. 2, while the % moisture content per DW is a scalar at each RH and can be seen in Fig. 1.

Supplementary Table 3: Model constants.

	Definition	Value and Units	Comments
A_{CM}	Cuticle area of one surface	7.85×10^{-5} m^2	
b	Thickness of cuticle	1.87×10^{-5} m	[2]
c_{min}	Initial concentration of water in a relatively dry cuticle, as a function of RH	mol/m^3	For [0, 0.387, 0.6, 0.7, 0.8, 0.9, 0.98, 0.999]RH, c_{min} =[0, 4515, 7168, 4515, 2596.1, 1140.9, 208, 300]
$c_{\text{H}_2\text{O}}^{\text{pure}}$	Pure water concentration as a function of temperature	mol/m^3	$\rho_{\text{H}_2\text{O}}/M_{\text{w}}$
c_∞	Atmospheric vapour concentration of air far from cuticle, as a function of temperature, T, in K	mol/m^3	$P_{\text{v}}\ H/(R\ T)$
$D_{\text{H}_2\text{O}}^{\text{bulk}}$	Self/bulk diffusion coefficient of water as a function of temperature	m^2/s	For temperatures [20, 25, 30, 35, 40, 45, 50] °C, [2.022, 2.296, 2.59, 2.919, 3.24, 3.575, 3.968]$\times 10^{-9}$ m^2/s[8,9]
DW	Total dry weight of isolated tomato fruit cuticle for experiment with humidity	137.7 mg	Calculated based on experimental data. This is the equivalent of using 85 individual cuticle discs for the experiment conducted over 6 hours
E	Activation energy for diffusion in the lipophilic pathway	2.64×10^4 Pa m^3/mol	For waxy isolated tomato fruit cuticles[10]
F_{s}	Fractal scaling dimension	1.075	Chosen to agree with experimental data
h	Moisture transfer coefficient	0.012 m/s	Chosen to agree with experimental data
H	Relative humidity as a fraction		$H = RH/100\% = p/p_{\text{sat}}$, p is the partial pressure of water vapour, p_{sat} is the pressure of saturated water vapour
k	Rate constant for water binding to cellulose on the cuticle surface	6.5×10^{-9} m/s	Chosen to agree with experimental data
k_1	Rate constant for water binding to cellulose on the outside cuticle surface	m/s	$k_1 = a_{\text{surf}}\ k_2$, where $a_{\text{surf}} = 0.1$
k_2	Rate constant for water binding to cellulose on the inside cuticle surface	m/s	$k_2 = W_C\ k$, where W_C is defined around Supplementary Equation (5)[5] as a function of relative humidity
K	GAB isotherm constant	0.46	[11]
L	Control volume length	1 m	
M_{w}	Molecular weight water	18.015 g/mol	
N	Avogadro constant	6.022×10^{23} mol^{-1}	
n	Number of tomato cuticle discs used in timed experiment over 10 minutes	1	Calculated based on experimental data[2]
P_{v}	Saturated water vapour pressure in air as a function of temperature in °C, $T_C = 20$°C	Pa	$611.21 \exp[(18.678 - T_C/234.84)(T_C/(273.15 + T_C))]$
R	Gas constant	8.31 Pa·m^3/K/mol	
$r_{\text{H}_2\text{O}}$	Van der Waals radius of a water molecule	1.5×10^{-10} m	
$r_{\text{A}}^{\text{max}}$	Maximum radius of aqueous pores	2.12×10^{-9} m	For tomato fruit cuticle,[12]
T	Temperature	K	
β	Langmuir parameter	m^3/mol	Equilibrium parameter of adsorbed water
β_{C}	GAB parameter	4	Equilibrium parameter of adsorbed water for GAB isotherm[11]
ε_{L}	Lipophilic pathway porosity	0.03	[13]
ε_{C}	Cellulose porosity	0.265	Chosen to agree with experimental data
η_{A}	Density of aqueous pores in cuticle	2.18×10^{15} m^{-2}	[14]
Γ_{S}	Langmuir saturation constant	mol/m^2	$0 < \Gamma_{\text{A}} < \Gamma_{\text{S}}$, saturation concentration of water adsorbed per unit area in the aqueous pathway
Γ_{SC}	GAB constant	6663 mol/kg	Monolayer concentration of water adsorbed per volume cellulose[11]
$\rho_{\text{H}_2\text{O}}$	Liquid density of water as a function of temperature	g/m^3	$(999.848 + 6.338 \times 10^{-2} T_C - 8.524 \times 10^{-3} T_C^2 + 6.943 \times 10^{-5} T_C^3 - 3.821 \times 10^{-7} T_C^4)1000$[15]
ρ_{C}	Density of cellulose fibres	1450 kg/m^3	[11]

Supplementary Note 3: Additional Sensitivity Analysis Results

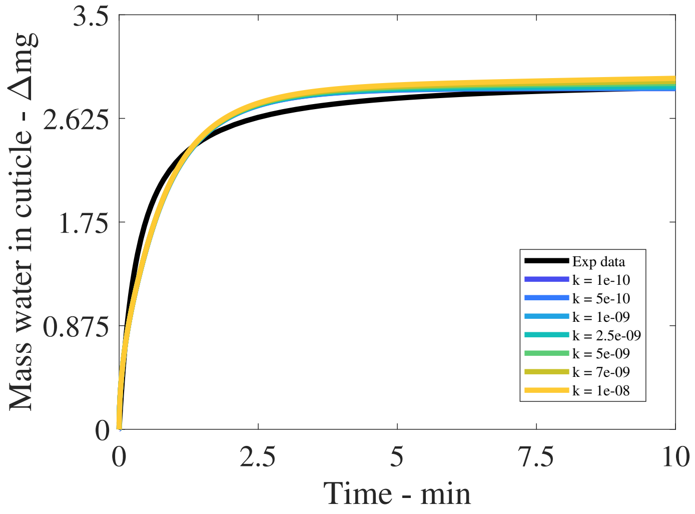

Supplementary Figure 2: Sensitivity analysis of the model, compared to the results for validation (v) and the experimental data (Exp data in black) as a reference point, all performed at 60%RH. All model parameters are kept constant except the sensitivity parameter k, the rate coefficient for cellulose binding on the boundaries.

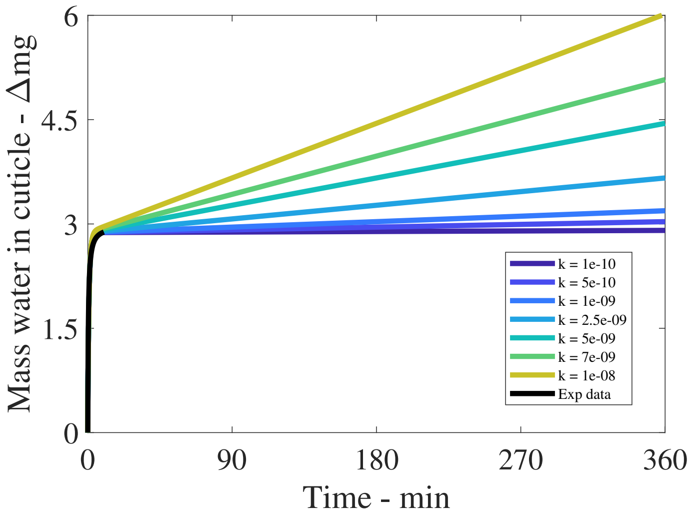

Supplementary Figure 3: Sensitivity analysis of the model, compared to the results for validation (v) and the experimental data (Exp data in black) as a reference point, all performed at 60%RH. All model parameters are kept constant except the sensitivity parameter k, the rate coefficient for cellulose binding on the boundaries.

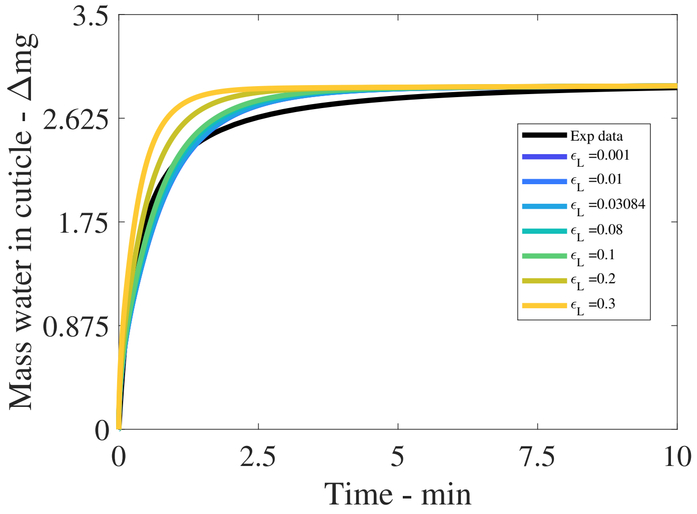

Supplementary Figure 4: Sensitivity analysis of the model, compared to the results for validation (v) and the experimental data (Exp data in black) as a reference point, all performed at 60%RH. All model parameters are kept constant except the sensitivity parameter for the porosity of the lipophilic pathway, ε_L.

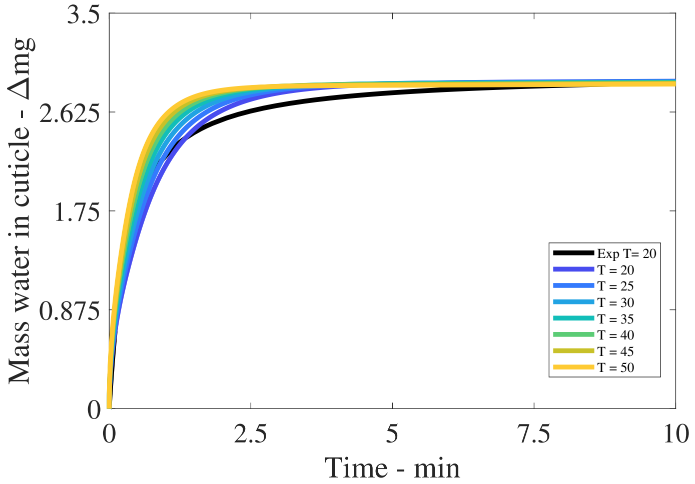

Supplementary Figure 5: Sensitivity analysis of the model, compared to the results for validation (v) and the experimental data (Exp data in black) as a reference point, all performed at 60%RH. All model parameters are kept constant except the sensitivity parameter for temperature, T.

References

[1] Luque, P., Gavara, R. & Heredia, a. A study of the hydration process of isolated cuticular membranes. *New phytologist* **129**, 283–288 (1995). URL `http://onlinelibrary.wiley.com/doi/10.1111/j.1469-8137.1995.tb04298.x/abstract`.

[2] Chamel, A., Pineri, M. & Escoubes, M. Quantitative determination of water sorption by plant cuticles. *Plant, Cell and Environment* **14**, 87–95 (1991).

[3] Coret, J. M. & Chamel, A. R. Influence of some nonionic surfactants on water sorption by isolated tomato fruit cuticles in relation to cuticular penetration of glyphosate. *Pesticide Science* **38**, 27–32 (1993).

[4] Tredenick, E. C., Farrell, T. W., Forster, W. A. & Psaltis, S. T. P. Nonlinear porous diffusion modeling of hydrophilic ionic agrochemicals in astomatous plant cuticle aqueous pores: A mechanistic approach. *Frontiers in Plant Science* **8**, 746 (2017). URL `http://journal.frontiersin.org/article/10.3389/fpls.2017.00746`.

[5] Dominguez, E. & Heredia, A. Water hydration in cutinized cell walls: a physico-chemical analysis. *Biochimica et Biophysica Acta (BBA)-General Subjects* **1426**, 168–176 (1999). URL `http://www.sciencedirect.com/science/article/pii/S0304416598001524`.

[6] Schieferstein, R. & Loomis, W. Development of the cuticular layers in angiosperm leaves. *American Journal of Botany* **46**, 625–635 (1959). URL `http://www.jstor.com/stable/2439666`.

[7] Heredia-Guerrero, J. A. *et al.* Infrared and raman spectroscopic features of plant cuticles: a review. *Frontiers in Plant Science* **5**, 305 (2014).

[8] Mills, R. Self-diffusion in normal and heavy water in the range 1-45. deg. *Journal of Physical Chemistry* **77**, 685–688 (1973).

[9] Easteal, A. J., Price, W. E. & Woolf, L. A. Diaphragm cell for high-temperature diffusion measurements. tracer diffusion coefficients for water to 363 k. *Journal of the Chemical Society, Faraday Transactions 1: Physical Chemistry in Condensed Phases* **85**, 1091–1097 (1989).

[10] Knoche, M., Petracek, P. D., Bukovac, M. J. & Shafer, W. E. Urea penetration of isolated tomato fruit cuticles. *Journal of the American Society for Horticultural Science* **119**, 761–764 (1994).

[11] Bedane, A. H., Eić, M., Farmahini-Farahani, M. & Xiao, H. Theoretical modeling of water vapor transport in cellulose-based materials. *Cellulose* **23**, 1537–1552 (2016).

[12] Schreiber, L. & Schönherr, J. *Water and Solute Permeability of Plant Cuticles: Measurement and Data Analysis*, vol. 616 (Springer, 2009).

[13] Tredenick, E. C., Farrell, T. W. & Forster, W. A. Mathematical modelling of hydrophilic ionic fertiliser diffusion in plant cuticles: Lipophilic surfactant effects. *Plants* **8** (2019). URL `https://www.mdpi.com/2223-7747/8/7/202`.

[14] Tredenick, E. C., Farrell, T. W. & Forster, W. A. Mathematical modeling of diffusion of a hydrophilic ionic fertilizer in plant cuticles: Surfactant and hygroscopic effects. *Frontiers in Plant Science* **9**, 1888 (2018). URL `https://www.frontiersin.org/article/10.3389/fpls.2018.01888`.

[15] Jones, F. E. & Harris, G. L. Its-90 density of water formulation for volumetric standards calibration. *Journal of research of the National Institute of Standards and Technology* **97**, 335 (1992).
